# Magnetic resonance imaging of sacroiliitis in children: frequency of findings and interobserver reliability

**DOI:** 10.1007/s00247-018-4185-x

**Published:** 2018-07-09

**Authors:** Katharine E. Orr, Savvas Andronikou, Marc James Bramham, Izidora Holjar-Erlic, Flavia Menegotto, Athimalaipet V. Ramanan

**Affiliations:** 1 Bristol Royal Hospital for Children, University Hospitals Bristol NHS Foundation Trust, 24 Upper Maudlin Street, Bristol, BS2 8BJ UK; 2 Peninsula Radiology Academy, Plymouth International Business Park, Plymouth, UK; 3 University Hospitals Bristol NHS Foundation Trust, Bristol, UK

**Keywords:** Children, Interobserver variability, Magnetic resonance imaging, Sacroiliitis, Sacroiliac joint

## Abstract

**Background:**

Clinicians increasingly rely on imaging in juvenile idiopathic arthritis (JIA) to identify sacroiliitis and guide treatment. However, there is limited evidence about magnetic resonance imaging (MRI) for sacroiliitis in children, and interobserver reliability is variable.

**Objective:**

Identify the frequency of MRI findings in children with suspected sacroiliitis, calculate inter-reporter reliability and assess the value of diffusion-weighted imaging and contrast-enhanced sequences.

**Materials and methods:**

We retrospectively reviewed 3 years of sacroiliac joint MRI records for suspected sacroiliitis in patients <21 years at a United Kingdom tertiary referral paediatric hospital. Five radiologists (panel of three radiologists and two independent radiologists) reviewed all MRI examinations using a pictorial checklist to identify oedema, effusions, diffusion-weighted signal abnormality, enhancement, erosions and sclerosis. The frequency of panel findings was reported. Interobserver agreement was calculated using the Cohen kappa coefficient.

**Results:**

An MRI diagnosis of sacroiliitis was made in 12 of 99 examinations (12%). The findings in all scans included oedema (9%), erosions (8%), diffusion-weighted signal abnormality (6%), abnormal enhancement (6%) and effusion (4%). All scans with abnormal contrast enhancement had other MRI features of sacroiliitis. Interobserver agreement was slight to moderate.

**Conclusion:**

Oedema and erosions were the most common findings. Inter-reporter reliability was variable with at best moderate agreement for the presence of sacroiliitis and erosions. The use of contrast enhancement for diagnosing sacroiliitis in children with JIA may be questionable.

## Introduction

Sacroiliitis affects 30% of children with the enthesitis-related arthritis subtype of juvenile idiopathic arthritis (JIA) [1]. Typically, patients with juvenile spondyloarthritis present with lower limb arthritis and enthesitis, with sacroiliitis occurring later [2, 3]. The diagnosis can be challenging. Inflammatory back pain is a late feature in children who can be asymptomatic and examination findings are unreliable [3, 4]. However, sacroiliitis causes reduced mobility and disability [5], with worse functional status in those presenting as children [3]. Axial disease responds poorly to conventional treatments [5–10]. Anti-tumour necrosis factor drugs as first-line agents for sacroiliitis increase mobility, improve quality of life and reduce pain [3, 6–10]. Increasingly, clinicians rely on imaging in JIA to identify those with axial disease who would benefit from biologics, highlighting the importance of quality imaging and reliable reporting to allow correct patient selection.

The complex structure and orientation of the sacroiliac joints pose challenges for imaging. Interpretation is more challenging in the immature skeleton. Radiographs and computed tomography (CT) are now obsolete for sacroiliac joint imaging [4, 5, 11, 12]. Magnetic resonance imaging (MRI) is now the modality of choice as it can demonstrate active inflammation, seen long before structural changes develop [12].

Acute and chronic manifestations of sacroiliitis can be seen on MRI. Acute or active findings include bone marrow oedema, effusions and synovitis [12]. Fluid within the joint (effusion) can be physiological in adults, but in children some report this as evidence of synovitis [2, 13]. High T2-weighted signal intensity or enhancement of the capsule, insertions of ligaments or tendons represents capsulitis or enthesitis, respectively [12]. Chronic/structural findings include erosions, sclerosis, fat deposition and ankylosis. Periarticular fat deposition in spondyloarthropathy represents areas of previous inflammation after biological treatment [12]. Despite clinicians’ reliance on MRI for diagnosing sacroiliitis, the evidence in children is limited [2, 14].

There are no reporting standards or diagnostic criteria for paediatric sacroiliac MRI. Most paediatric imagers adapt adult scoring systems [4, 5], but these are not validated in children [15]. Inter-reporter reliability is variable [4, 14, 16]. MRI is often used as the gold standard for diagnosis [4, 16]. This may be justified as MRI can be superior to clinical modes of assessment in children, but definitive histopathological correlation is rarely available. There is a mismatch between the reliance on MRI for diagnosis and the lack of evidence to support current imaging and reporting practice.

Our aim was to review the imaging findings of sacroiliac joint MRIs in children with suspected sacroiliitis to determine the presence and distribution of features of sacroiliitis and determine the reliability of each MRI finding. A further aim was to evaluate the additional value of diffusion-weighted imaging (DWI) and contrast-enhanced sequences with regard to the presence and distribution of disease and reliability of the detection.

## Materials and methods

### Study type and patient selection

A retrospective descriptive study was performed reviewing all those referred to a children’s tertiary referral hospital imaging department for sacroiliac joint MRI for suspected sacroiliitis over a 3-year period (August 2013–June 2016). Ethical approval was waived. Referral criteria for MRI investigation for suspected sacroiliitis at this institution were teenagers presenting with back pain or children of any age with known JIA and back pain. All patients younger than 21 years who had undergone MRI of the sacroiliac joints for possible sacroiliitis were included. Any incomplete or irretrievable studies were excluded.

### MRI technique

All MRI scans were performed in the outpatient setting on a 3-T scanner (Skyra; Siemens, Erlangen, Germany) using the standard departmental protocol, which included coronal oblique short tau inversion recovery (STIR) spin echo (matrix 256 × 256, 3-mm slice thickness, TR 4,000 ms, TE 36 ms, TI 200 ms), T1-weighted spin echo non-fat-saturated (matrix 320 × 320, slice thickness 3 mm, TR 679 ms, TE 12 ms), T1-weighted spin echo fat-saturated post-contrast (matrix 320 × 320, slice thickness 3 mm, TR 680 ms, TE 12 ms), and diffusion-weighted sequences at b-values of 50, 400 and 800 (matrix 140 × 140, slice thickness 3 mm, TR 4,500 ms, TE 79 ms). Apparent diffusion coefficient (ADC) maps were calculated by the scanner. Diffusion-weighted sequences were reconstructed from the axial images and read with inverted grey scale in the axial and coronal oblique planes.

### Reporting design

All eligible MRI examinations were retrospectively reviewed by three readers: Reader 1 was a panel of three radiologists (a pediatric radiology consultant with 20 years experience and two 4th year radiology registrars); Reader 2 was a pediatric radiology consultant with 8 years of experience (8 in total, 5 as a paediatric radiologist); and Reader 3 was a pediatric radiology consultant with 10 years of paediatric radiology experience. All readers were blinded to each other (except for the panel, whose members reported together), the original radiology reports and any previous imaging. Readers were not blinded to the clinical history of possible juvenile spondyloarthritis due to the known standard referral practice. Reporters were given guidance on how to complete the reporting pro forma before commencing the study.

### Reporting pro forma

A pictorial pro forma was designed in the style of a checklist for each sign using a schematic of the sacroiliac joints to aid systematic reporting. This was created with reference to the features of sacroiliitis reported in the literature, including acute and chronic findings according to the sequence each finding should be sought on [2]. The reporting pro forma was used to record the presence of bone marrow oedema, effusion, erosion, fatty proliferation, sclerosis, ankylosis, diffusion-weighted signal abnormality (increased diffusion indicating oedema) and abnormal enhancement at both joints and with regard to the craniocaudal position as well as the iliac vs. sacral side of the joint.

### Definition of MRI findings for reporting (Table 1)

**Table 1:** Definitions of magnetic resonance imaging findings

Bone marrow oedema	Regions of periarticular/subchondral high T2-weighted and low T1-weighted signal.
Effusion	Fluid or high T2-weighted signal within the sacroiliac joint.
Diffusion-weighted imaging abnormality	High signal on diffusion-weighted images with corresponding high apparent diffusion coefficient either at sites of bone marrow oedema or effusion.
Enhancement	Enhancement of the subchondral regions was defined as osteitis. Enhancement of the synovium was defined as synovitis.
Erosion	Low T1-weighted signal in subchondral regions with either corresponding low or high T2-weighted signal (depending on the presence of active inflammation); irregular indentations of the articular surface at the synovial part of the joint.
Sclerosis	Low T1- and T2-weighted signal bands in the periarticular regions.
Ankylosis	Periarticular low signal on all sequences with blurring/disappearance of the joint margins.

Bone marrow oedema was defined as the presence of regions of subchondral or periarticular high T2-weighted signal with corresponding low T1-weighted signal (and with increased diffusion, if noted). Abnormal enhancement was defined as either osteitis when seen within the subchondral or periarticular regions or synovitis with enhancement of the synovium. Effusions were defined as fluid/high T2-weighted signal within the sacroiliac joint. Signal abnormality on DWI with corresponding high apparent diffusion coefficients (ADC) in the periarticular regions or within the joint itself was reported as “free diffusion” related to either bone marrow oedema or joint effusion. Any regions of restricted diffusion (increasing signal with increasing b-value and corresponding low ADC) were also noted if seen in relation to the presence of red marrow or in the case of other pathology.

Erosions were defined as irregular indentations of the articular surface at the synovial portion of the sacroiliac joint, with corresponding low T1-weighted signal in the subchondral/periarticular regions and either with corresponding low or high T2-weighted signal depending on the presence of active inflammation. Care was taken not to call the fibrous portion of the joint abnormal. Subchondral sclerosis was defined as low T1- and T2-weighted signal bands in the periarticular regions. Periarticular fat deposition was defined as regions of high signal on conventional T1-weighted signal in the subchondral regions. Ankylosis was defined as periarticular low signal on all sequences, with blurring/disappearance of the joint margins.

### Statistical analysis

Overall frequency of findings was reported according to the panel read. Interobserver reliability was calculated for the overall presence of sacroiliitis and for each sign using both percentage agreement and the Cohen kappa coefficient.

## Results

**Fig. 1:**
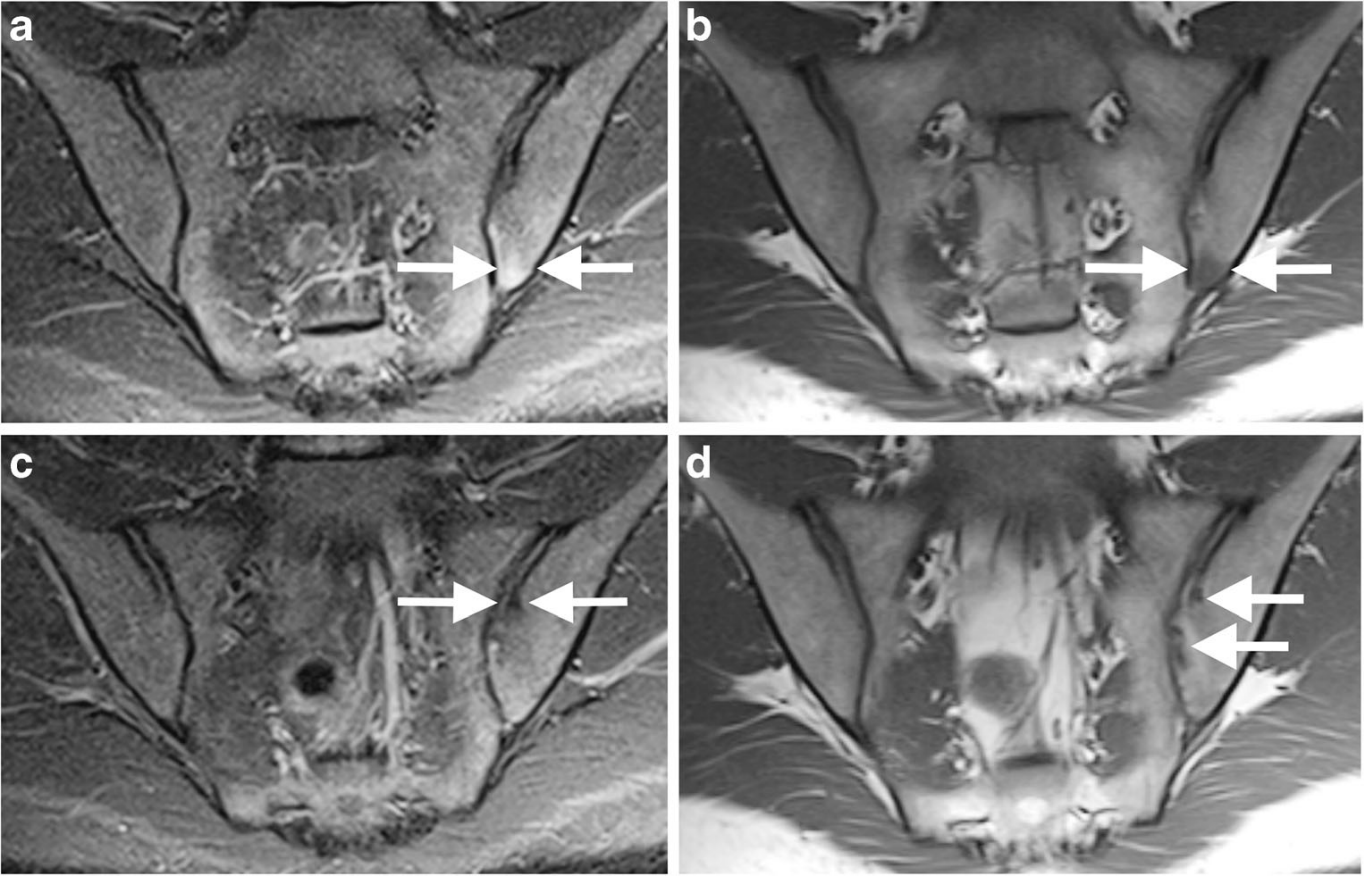
A 15-year-old girl with reactive arthritis and clinical sacroiliitis. **a-b** Coronal oblique short tau inversion recovery (STIR) spin echo (TR 4,000 ms, TE 36 ms) (**a**) and T1-weighted spin echo (TR 679 ms, TE 12 ms) (**b**) images demonstrate oedema on the iliac side of the inferior left sacroiliac joint (*arrows*), seen as high signal in (**a**) STIR and low signal in (**b**). **c-d** Coronal oblique STIR spin echo (**c**) and T1-weighted spin echo (**d**) images at a different location show broad-based erosions (*arrows*) more superiorly on the iliac side of the left sacroiliac joint, better appreciated in (**d**)

**Fig. 2:**
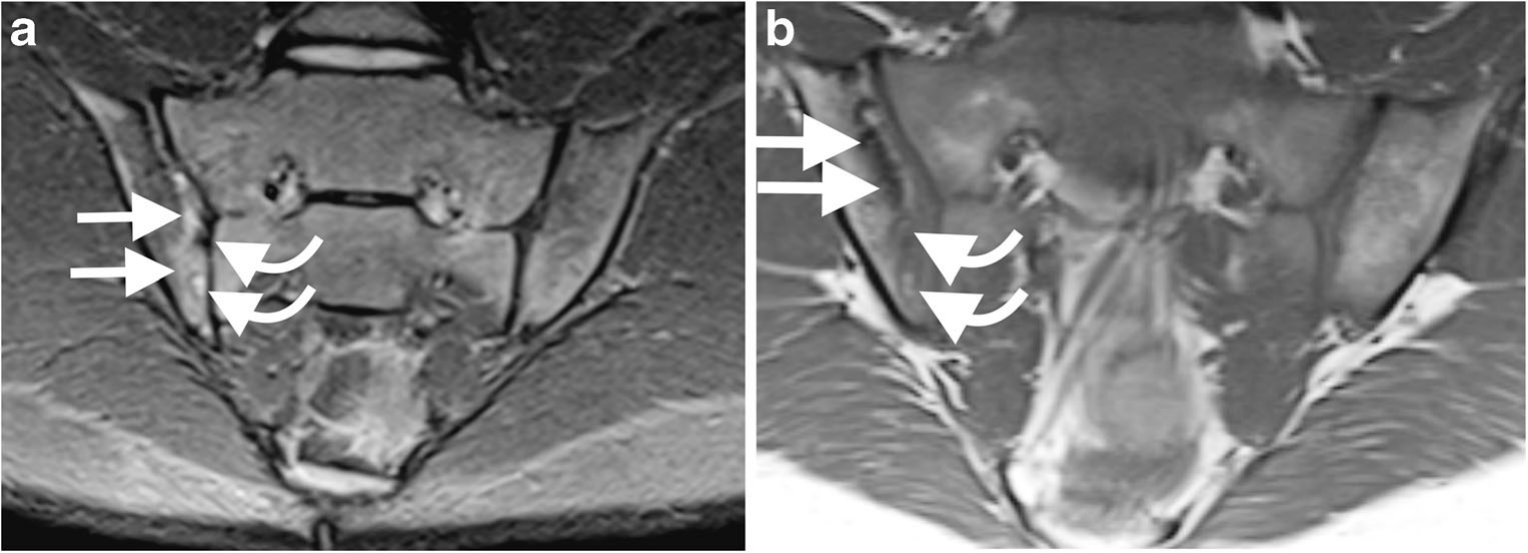
A 17-year-old boy with juvenile spondyloarthritis. **a-b** Coronal oblique short tau inversion recovery (STIR) spin echo (TR 4,000 ms, TE 36 ms) (**a**) and T1-weighted spin echo (TR 679 ms, TE 12 ms) (**b**) images at slightly different locations in the joint demonstrate multiple erosions on the iliac side of the right sacroiliac joint (*curved arrows*) with surrounding oedema (*straight arrows*), which is high signal on (**a**) and low signal on (**b**)

**Fig. 3:**
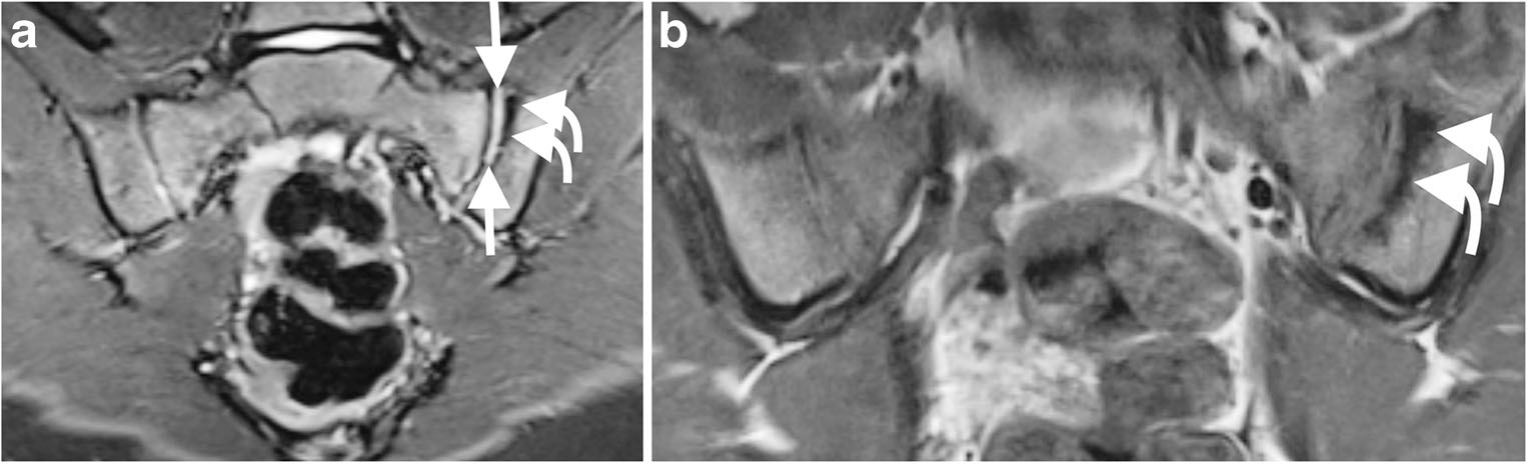
A 9-year-old girl with juvenile idiopathic arthritis and suspected sacroiliitis. **a** Coronal oblique short tau inversion recovery (STIR) spin echo (TR 4,000 ms, TE 36 ms) image demonstrates a left sacroiliac joint effusion, seen as high signal within the joint (*straight arrows*) and subchondral sclerosis (*curved arrows*). **b** T1-weighted spin echo (TR 679 ms, TE 12 ms) images also demonstrate sclerosis adjacent to the left sacroiliac joint, particularly on the iliac side of the joint (*curved arrows*)

**Fig. 4:**
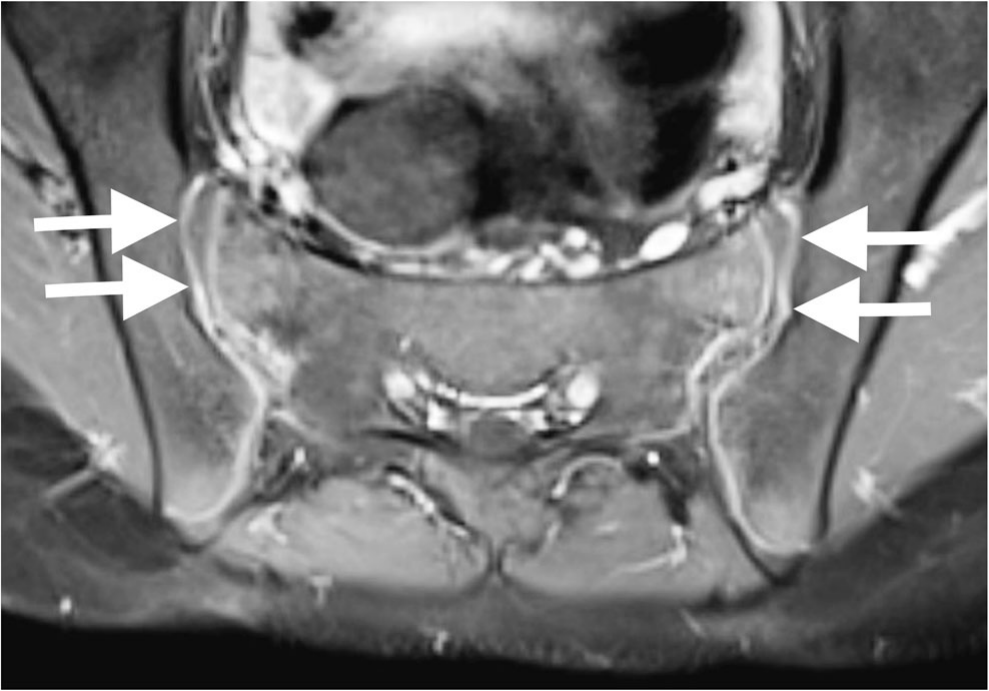
A 12-year-old girl with mechanical back pain. **a** Contrast-enhanced T1-weighted image (TR 679 ms, TE 12 ms) demonstrates smooth synovial enhancement (*arrows*) of both sacroiliac joints in the absence of other findings. The authors believe this may be a normal finding

One hundred and two MRI examinations had been performed and were eligible for the study. Three were excluded as incomplete studies. This resulted in the inclusion of 99 MRI examination performed in 88 patients. Eleven examinations were repeat studies in patients who had already been scanned within the study period. These patients were not excluded and were reported independently (not in comparison to the initial study) but were noted by the primary investigator so that their impact on final results could be considered. The mean patient age was 15 years (range: 6–20 years, median: 15 years). Forty-one patients were female (47%). Examples of findings are demonstrated in Figs. 1, 2, 3 and 4.

### Sacroiliitis by consensus

The panel identified changes of sacroiliitis in 12 of 99 MRI examinations, giving an overall frequency of 12%. Of note, although one of these patients had two examinations in our date range, only the later MRI was positive, so there were no duplicated patients in the positive group. This group with positive MRI scans had a mean age of 15 years (range: 9–19 years, median: 14 years). Ten (83%) had unilateral changes (50% right, 50% left) and 2 (17%) had bilateral disease. There was a slight female predominance overall (7 patients, 58%) and both patients with bilateral disease were female. The frequency of findings is summarised in Table 2.

**Table 2:** Frequency (percentage of all 99 scans) of magnetic resonance imaging findings according to the panel (reader 1) and compared with the independent readers 2 and 3

	Reader 1 (panel of 3 radiologists)	Reader 2	Reader 3
Final diagnosis of sacroiliitis	12 (12%)	19 (19%)	39 (39%)
Bone marrow oedema	8 (8%)	19 (19%)	39 (39%)
Effusion	4 (4%)	2 (2%)	43 (43%)
Diffusion-weighted signal abnormality	5 (5%)	11 (11%)	11 (11%)
Enhancement	5 (5%)	15 (15%)	21 (21%)
Erosion	8 (8%)	7 (7%)	31 (31%)
Sclerosis	6 (6%)	1 (1%)	9 (9%)

### Overall frequency of findings suggesting inflammation

Eight patients had bone marrow oedema (8%) (right-side in four patients and left-side in four patients). Bone marrow oedema affected the sacral side of the sacroiliac joint in three cases, the iliac side in three cases and both sides of the joint in two cases.

Increased diffusion was seen in five patients (5%). This was right-side in three patients and left-side in three patients.

Abnormal enhancement was seen in five patients (5%) (right-side in two patients and left-side in three patients). While three patients had synovial enhancement and three had enhancement of the sacral aspect of the sacroiliac joint (osteitis), there were no cases of enhancement on the iliac aspect of the joint.

On the five examinations with abnormal enhancement, one showed synovial enhancement, two showed both synovial enhancement and osteitis in association with oedema, two had erosive changes and two had osteitis in association with oedema and erosive changes. Of note, the patient with enhancement only of the synovium also had a joint effusion.

Four patients had a sacroiliac joint effusion (4%) (three were left-side and one patient had bilateral effusions).

### Overall frequency of chronic/structural abnormality

All patients with structural findings had concomitant findings of active disease. Eight patients had erosions (8%), which were right-side in three patients and left-side in five patients. Two patients had erosions only on the iliac side of the sacroiliac joint, four had erosions only on the sacral side of the iliac joint and two patients had erosions on both iliac and sacral sides of the joint.

Sclerosis was seen in six patients (6%), which was right-side in two patients, left-side in two patients and bilateral in two patients. The sclerosis was seen on the iliac side of the joint in three patients, on the sacral side in one patient and on both sides of the joint in two cases.

### Interobserver agreement

Interobserver agreement is summarised in Table 3. Due to the low frequency of positive scans, reliability using Cohen kappa was believed to be more accurate, but percentage agreement was also calculated and is included in Table 3. Comparing the panel (reader 1) to the first independent observer (reader 2), the highest concordance assessed by Cohen kappa was for the composite variable sacroiliitis (κ=0.51, 95% confidence interval [CI] 0.27–0.72) and for erosions (κ=0.5, 95% CI could not be calculated). The concordance between the panel (reader 1) and the second independent observer (reader 3) was poorer (κ≤0.33).

**Table 3:** Interobserver Cohen kappa and proportion of agreement for overall diagnosis of sacroiliitis and for individual sign of sacroiliitis

	Reader 1 (panel) and reader 2			Reader 1 (panel) and reader 3		
	Percentage agreement	Cohen κ	95% confidence interval	Percentage agreement	Cohen κ	95% confidence interval
Sacroiliitis	87%	0.51	0.27–0.72	72%	0.31	0.15–0.48
Bone marrow oedema	85%	0.38	0.12–0.61	75%	0.29	0.12–0.45
Erosion	93%	0.5	0.12–0.79	82%	0.33	0.14–0.51
Effusion	96%	0.32	N/A	60%	0.06	−0.02-0.16
Diffusion-weighted signal abnormality	88%	0.2	−0.06–0.50	90%	0.33	−0.02–0.62
Enhancement	86%	0.3	0.01–0.56	82%	0.28	0.05–0.51
Sclerosis	94%	0.24	0.00–0.58	88%	0.19	−0.08–0.48

## Discussion

In this explorative study, 12 cases out of 99 (12%) were considered to have sacroiliitis based on the panel’s MRI findings, a lower percentage than the 20–31% reported elsewhere in the literature [2, 16]. This may be due to different imaging referral practices/demographics. One study reported a higher frequency (64%), possibly due to referral bias or MRI overdiagnosis (due to poor imaging technique, no diagnostic criteria or subjectivity of interpretation, as demonstrated in our study by reader 3’s findings (39% sacroiliitis) [4].

Bone marrow oedema (Figs. 1 and 2) was seen in 8 cases (8%), fewer than the 20% reported in other studies [2, 16]. This may be due to different referral practices, e.g., including those with a diagnosis [16], as opposed to those with back pain.

Use of contrast medium must be justified, especially given concerns about intracranial gadolinium deposition [17–19]. Abnormal enhancement was seen in 5% of cases, but these all had other features of active sacroiliitis. Giving contrast did not identify any additional cases. Some authors say contrast is essential to identify synovial enhancement, some saying this can be the only evidence of synovitis, but they do not report effusions [4, 5]. Others, similar to this study, report that all cases with synovial enhancement also have effusions [2, 13]. Furthermore, it is unknown if isolated synovial enhancement is pathological. Those advocating contrast state that normal paediatric sacroiliac joints do not enhance [2]. However, the quoted paper on normal sacroiliac joints in children states that capsular enhancement is normal, with no specific comment on the synovial portion [20]. In adults, synovitis alone is not sufficient to diagnose sacroiliitis [12]. Some say synovitis can be a solitary finding in children, but this is not proven. We believe a thin rim of synovial enhancement may be normal in children (Fig. 4), but this is difficult to prove. Despite this, discounting those not reporting effusions, the literature shows that all cases with enhancement have other MRI signs of sacroiliitis, which refutes the use of contrast in children.

DWI is increasingly used. Inflammatory changes in sacroiliitis are high signal on DWI and demonstrate free diffusion. Only 5% of MRIs in this study had abnormal diffusion-weighted signal. Of note, three patients with oedema and four with effusion had no corresponding DWI abnormality. This could be due to lower resolution of images or artefact. One group found significantly higher ADC values in sacroiliitis and advocate diffusion to quantify severity of inflammation in enthesitis-related arthritis [21]. However, they also found that ADC values in skeletally immature controls overlap with sacroiliitis [22]. Others use ADC values to assess response to anti-tumour necrosis factor treatment [23]. In this study, all cases with abnormal diffusion-weighted signal had high signal on STIR. However, we found that DWI can differentiate periarticular high signal due to red marrow (can exhibit restricted or free diffusion and is iso- or hyperintense to muscle on T1-weighted images) from bone marrow oedema (exhibits free diffusion and is hypointense to muscle on T1-weighted images). For these reasons, and as DWI is safe, we believe it should be included in protocols.

The frequency of erosions (8%) (Figs. 1 and 2) and sclerosis (6%) (Fig. 3) is surprising as structural changes are uncommon in children [2]. One explanation is that the positive cohort was older (mean: 15 years, range: 9–19 years, median: 14 years) compared to the total group (mean: 15 years, range: 6–20 years, median: 15 years). However, the youngest patient with erosions and sclerosis was 9 years old. Some say structural changes are uncommon until late teenage years [2, 15]; others report a higher frequency of 56% [4]. In our study, aside from a case with unilateral sclerosis, all cases with structural changes also had oedema and/or effusion, supporting beliefs that children with structural change usually still have active disease [15]. However, there is a reported case of an erosion without coexisting oedema or effusion, highlighting the need for paediatric diagnostic criteria [16]. In adults, structural changes are not sufficient evidence of sacroiliitis [12]. The definition of a positive MRI diagnosis in children, mostly focused on acute signs, is disputed. Adding structural changes to diagnostic criteria may not alter the number of diagnoses and more research is needed.

Deducing sensitivity and specificity of MRI findings for sacroiliitis without a gold standard is problematic. One study reports that consensus agreement on the presence of sacroiliitis is most sensitive (55%), followed by synovial enhancement (52%), concluding a negative MRI cannot exclude sacroiliitis [2]. Using synovial enhancement as a sign of sacroiliitis is questionable. Most agree that structural changes are specific for sacroiliitis, but these often occur late. Oedema is nonspecific in adults [12, 24] but more specific in children [2], possibly due to a reduced likelihood of other pathology in children. Some suggest that oedema on a single image can represent sacroiliitis in children, in contrast to adults [15]. However, normal MRI appearances of the immature skeleton are not well known; foci of T2 hyperintensity can be seen elsewhere in the body [25], but the relevance of this phenomenon in the sacroiliac joints is uncertain.

The most reliable MRI findings in our study were the overall presence of sacroiliitis and the identification of erosions, both demonstrating moderate interobserver agreement. The remainder of findings demonstrated poor agreement, ranging from slight to fair (Table 3). The poor concordance of findings between Reader 1 and Reader 3 emphasizes that reporting these MRIs is difficult and interpretation is subjective. The literature reports variable inter-reader reliability. Some report moderate to substantial agreement [1, 16]. One group found poor agreement on composite scores and individual signs but substantial agreement on the presence of sacroiliitis [4], possibly reflecting their high frequency of positive scans.

Some use MRI as the gold standard for diagnosis [4, 16], but no papers can prove the MRI features represent true pathology. There are conflicting opinions on the reporting and the significance of findings. In addition, some discuss imaging findings but show images where pathology is not appreciated or with pitfalls presented as pathology. Finally, there is a tendency for paediatric studies to apply adult literature to children, but sacroiliac joint imaging is more difficult in children given their smaller size, and increased cartilage and red marrow [4]. Also, structural changes commonly seen in adults are not predominant in children. These factors, combined with no diagnostic criteria and variable inter-reporter agreement make interpreting the evidence difficult. Given the lack of a definitive means of diagnosis and that MRI is often used as the gold standard, more emphasis needs to be placed on the reliability of MRI findings.

There is limited discussion about the need for more standardised reporting. As these MRIs are frequently performed in patients with JIA with no symptoms of sacroiliitis, radiologists’ reports influence management decisions. We suspect that current practice is to overcall findings and overdiagnose sacroiliitis on MRI in children. Using a pictorial reporting pro forma, which categorizes MRI features of sacroiliitis by the appropriate sequence as used in this study may help improve consistency in reporting and would allow internal and external inter-reader agreement in greater numbers of reporters across hospitals. In the absence of a gold standard, radiologists must improve reliability based on agreed criteria. In addition, consensus and expert groups should create strict imaging definitions to improve agreement.

### Limitations

Despite a 3-year study period, there was a low number of positive MRI cases. However, to the best of our knowledge, the patient cohort was one of the largest in the literature. The low frequency of cases reflects the expected frequency of sacroiliitis in this patient group. The Cohen kappa was used to calculate interobserver agreement. Using the Randolph free marginal kappa to account for the low frequency of positive cases was considered, but it was decided that agreement on large numbers of negative scans would falsely increase interobserver agreement. Finally, the retrospective nature of the study and lack of clinicopathological correlation with MRI findings is a universal limitation of determining the accuracy of MRI for diagnosing sacroiliitis. As there is no gold standard for diagnosis, the possibility of overdiagnosis in this study is also possible. MRI findings were not correlated with clinical findings because these are considered unreliable and would not be standardised as part of a retrospective study. These factors make determination of reliability of MRI findings even more important.

## Conclusion

Bone marrow oedema and erosions were the most common findings of sacroiliitis in children. Reliability of MRI signs of sacroiliitis in children is at best moderate for overall impression and presence of erosions with lower agreement for the active signs of sacroiliitis. Contrast is not required in routine sacroiliac joint MRI in children with JIA because enhancement does not occur without other features. More standardised reporting and paediatric diagnostic criteria are required to improve the quality of evidence in the future, using a reporting checklist may help with this.
